# Tissue Inhibitor of Metalloproteinases-1 Interacts with CD74 to Promote AKT Signaling, Monocyte Recruitment Responses, and Vascular Smooth Muscle Cell Proliferation

**DOI:** 10.3390/cells12141899

**Published:** 2023-07-20

**Authors:** Simon Ebert, Lan Zang, Noor Ismail, Michael Otabil, Adrian Fröhlich, Virginia Egea, Susann Ács, Mikkel Hoeberg, Marie-Luise Berres, Christian Weber, José M. A. Moreira, Christian Ries, Jürgen Bernhagen, Omar El Bounkari

**Affiliations:** 1 Department of Vascular Biology, Institute for Stroke and Dementia Research, Klinikum der Universität München, Ludwig-Maximilian-University (LMU) Munich, 81377 Munich, Germanyjuergen.bernhagen@med.uni-muenchen.de (J.B.); 2 Institute for Cardiovascular Prevention (IPEK), Klinikum der Universität München, Ludwig-Maximilian-University (LMU) Munich, 80336 Munich, Germany; 3 Department of Drug Design and Pharmacology, Faculty of Health and Medical Sciences, University of Copenhagen, 2200 Copenhagen, Denmark; 4 Department of Internal Medicine III, RWTH Aachen University, 52074 Aachen, Germany; 5 Munich Cluster for Systems Neurology (SyNergy), 81377 Munich, Germany; 6 Munich Heart Alliance, 80802 Munich, Germany

**Keywords:** cytokine, chemokine, cell migration/adhesion, proliferation, vascular inflammation, atherogenesis

## Abstract

Tissue inhibitor of metalloproteinases-1 (TIMP-1), an important regulator of matrix metalloproteinases (MMPs), has recently been shown to interact with CD74, a receptor for macrophage migration inhibitory factor (MIF). However, the biological effects mediated by TIMP-1 through CD74 remain largely unexplored. Using sequence alignment and in silico protein–protein docking analysis, we demonstrated that TIMP-1 shares residues with both MIF and MIF-2, crucial for CD74 binding, but not for CXCR4. Subcellular colocalization, immunoprecipitation, and internalization experiments supported these findings, demonstrating that TIMP-1 interacts with surface-expressed CD74, resulting in its internalization in a dose-dependent manner, as well as with a soluble CD74 ectodomain fragment (sCD74). This prompted us to study the effects of the TIMP-1–CD74 axis on monocytes and vascular smooth muscle cells (VSCMs) to assess its impact on vascular inflammation. A phospho-kinase array revealed the activation of serine/threonine kinases by TIMP-1 in THP-1 pre-monocytes, in particular AKT. Similarly, TIMP-1 dose-dependently triggered the phosphorylation of AKT and ERK1/2 in primary human monocytes. Importantly, Transwell migration, 3D-based Chemotaxis, and flow adhesion assays demonstrated that TIMP-1 engagement of CD74 strongly promotes the recruitment response of primary human monocytes, while live cell imaging studies revealed a profound activating effect on VSMC proliferation. Finally, re-analysis of scRNA-seq data highlighted the expression patterns of TIMP-1 and CD74 in human atherosclerotic lesions, thus, together with our experimental data, indicating a role for the TIMP-1–CD74 axis in vascular inflammation and atherosclerosis.

## 1. Introduction

Atherosclerosis is a lipid-triggered chronic inflammatory disease of the arterial vessel wall and the main underlying condition of myocardial infarction, ischemic stroke, and peripheral artery disease, and thus, significantly contributes to morbidity and mortality worldwide [1]. Its initiation and progression involves various inflammatory processes that regulate the activation and recruitment of immune cells, which are key drivers of atherosclerotic plaque development [2]. Immune cell recruitment is stimulated and orchestrated by an array of cytokines and chemokines. Abundant evidence has been available for classical chemokines such as CCL2, CXCL1, CXCL2, CXCL9, CXCL10, CXCL11, and CXCL13, as well as the chemoattractant C5a [3,4,5,6], but more recently, a key role for non-classical mediators such as macrophage migration inhibitory factor (MIF) has emerged [7]. MIF is a long-known inflammatory cytokine and atypical chemokine [8,9,10], which drives atherogenic leukocyte recruitment through non-cognate interaction with the chemokine receptors CXCR2 and CXCR4 [9]. MIF engagement of its cognate receptor CD74, a type-II transmembrane glycoprotein also known as the plasma membrane form of MHC class II invariant chain or Ii [11,12,13] promotes cell proliferative responses and contributes to a variety of additional effects of MIF on vascular inflammation [11,12].

Tissue inhibitor of metalloproteinase-1 (TIMP-1) is a multifunctional protein and a member of the TIMP family of proteins, which also includes TIMP-2, -3, and -4. TIMPs act as key regulators of matrix metalloproteinases (MMPs), enzymes involved in extracellular matrix turnover [14]. TIMP-1 is widely expressed and secreted from various cell types, and its elevated levels are associated with chronic inflammatory diseases including cardiovascular diseases (CVDs) [15,16]. Besides its role as an endogenous inhibitor of MMPs, TIMP-1 also exhibits cytokine-like activities, which can directly influence cell behavior independent of its MMP-inhibiting activity [17,18,19]. TIMP-1 has been reported to bind to the surface receptors CD63 and LRP-1 [20,21], and most recently, CD74 has been suggested to act as an additional receptor for TIMP-1 [22,23,24]. TIMP-1–receptor interactions induce several intracellular signaling pathways to modulate cell behavior [18,20,21,25]. However, while TIMP-1 engagement of CD74 was found to induce internalization and signaling in lymphoma and breast cancer cells, the interaction between TIMP-1 and CD74 in immune and vascular cells has not been studied, and the impact of this ligand/receptor axis in vascular inflammation has remained unclear.

Here, we applied in silico docking and biochemical methods and confirmed that TIMP-1 is a ligand of CD74. We also demonstrated the binding of TIMP-1 to sCD74. Using colocalization and CD74 internalization experiments, we show that TIMP-1 engagement of CD74 extends to immune cells, i.e., monocytes. The phospho-kinase array and Western blotting experiments indicated that the AKT signaling pathway is activated following CD74 activation by TIMP-1 in monocytes. Transwell and 3D Chemotaxis experiments, as well as monocyte arrest studies under flow highlighted the role of this axis in leukocyte recruitment. IncuCyte-based live cell imaging to monitor VSMC proliferation pointed to an additional effect of TIMP-1–CD74 on vascular inflammation. Together with the identified TIMP-1 and CD74 expression patterns in re-analyzed scRNA-seq datasets from atherosclerotic plaques of carotid endarterectomy (CEA) patients, our study suggested a novel role for the TIMP-1–CD74 axis in vascular inflammation and atherosclerosis.

## 2. Materials and Methods

### 2.1. Isolation of Human Peripheral Blood Monocytes

Human peripheral blood mononuclear cells (PBMCs) were isolated as previously reported and in line with the local ethics requirements of LMU Munich University Hospital (ethics approval LMU Munich, Germany; AZ 18-104; 03/06/2018) [9]. Peripheral blood obtained from healthy donors was mixed (1:1) with phosphate-buffered saline (PBS) and added to Ficoll-Paque media solution (GE Healthcare, Freiburg, Germany), and PBMCs were separated by density gradient centrifugation using Ficoll-Paque Plus (Cytiva, MA, USA). Cells in the spongy layer were carefully removed and washed with PBS. Red blood cells (RBCs) were lysed using red blood cell lysis buffer (BioLegend, San Diago, CA, USA) for 3 min at room temperature (RT). Subsequently, cells were washed with RMPI 1640 media supplemented with 10% fetal bovine serum (FBS). Isolated PBMCs were cultured in RPMI 1640 media supplemented with 10% FBS and 1% penicillin/streptomycin (P/S) (Gibco, Karlsruhe, Germany) at 37 °C and 5% CO_2_. Finally, primary human monocytes were isolated by negative selection using the Pan Monocyte Isolation Kit, human (Miltenyi Biotec, Bergisch Gladbach, Germany), according to the manufacturer’s protocol.

### 2.2. Cell Culture and Cell Handling

THP-1 monocytic cells and primary PBMC-derived human monocytes were cultured in RPMI 1640 media + Glutamax supplemented with 10% FBS and 1% P/S (5000 U/mL). For serum-free cultivation of cells, FBS was replaced by 1% Nutridoma SP (Roche Applied Science, Mannheim, Germany) as described in [26]. Monomac 6 (MM6) cells were cultured in RPMI 1640 media + Glutamax supplemented with 10% FBS, 1% P/S (5000 U/mL), and 1% non-essential amino acids. Human aortic endothelial cells (HAoECs) were cultured in Endothelial Cell Basal Medium (EBM-2) (Lonza, Verviers, Belgium) supplemented with 1% penicillin/streptomycin and EGM™-2 SingleQuots^®^ supplements (Lonza). The cells were cultured at 37 °C with 95% humidity and 5% CO_2._

### 2.3. Pull down Assay

A 10× lysis buffer (Cell Signaling Technology, Danvers, MA, USA) containing 1% Triton-X 100, 1 mM PMSF, and half of a Pierce™ Protease Inhibitor tablet (ThermoFisher, Waltham, MA, USA) were prepared on ice and diluted with ddH2O up to a final concentration of 1× lysis buffer (referred to as lysis buffer). After transfection of HEK293 cells (16–24 h), the cells were lysed using 1.5 mL lysis buffer per Petri dish and incubated on a shaker for 15 min in a cold room at 4 °C. In the following step, the supernatant was collected (referred to as cell lysate) after a centrifugation step (20,000× *g* for 5 min) to remove the debris. Laemmli buffer was added to collect samples of the input prior to the following steps. First, only the cell lysate (500 μL) and anti-flag antibody (10 μL) were incubated on a rotating Eppendorf wheel at 4 °C for 1 h at a low speed of rotation to provide optimal conditions for the assembly of the protein complex. Afterwards, recombinant TIMP-1 (500 ng) was added to the solution and incubated on a rotating Eppendorf wheel at 4 °C for 1 h at a low speed of rotation overnight. The next day, 70 μL of magnetic protein G-coupled beads was washed twice with 500 μL lysis buffer after incubating the 1.5 mL Eppendorf tube on a DYNAL™ DynaMag™ Dynabeads™ DynaMag–2 magnet for 5 min each. Samples of the input were collected before the incubated solution was carefully added to the washed beads. After a 2 h incubation on a rotating Eppendorf at 4 °C at a low speed of rotation, Laemmli buffer was added to a sample of the unbound fraction. The beads were washed carefully 3 times with 500 μL lysis buffer on the DynaMag–2 magnet for 5 min each. Afterwards, 80 μL of lysis buffer was added to the beads. Finally, Laemmli buffer was added to the lysis buffer and beads. All collected samples were boiled for 10–20 min at 95 °C and spun down at 600 rpm (Thermal Shake lite, VWR, Radnor, PA, USA). Subsequently, the pulldown assay was analyzed by Western blotting using anti-FLAG (Cell Signaling, Cat#14793) and anti-TIMP1 (Abcam, Cat#ab61224, Cambridge, UK) antibodies. The pulldown assay involving recombinant TIMP-1 and the soluble CD74 ectodomain fragment (sCD74) was conducted using a consistent protocol. However, in this case, the pulldown was carried out with His-tagged Dynabeads (Invitrogen cat# 10103D, Waltham, MA, USA), recombinant human sCD74 (500 ng), and recombinant human TIMP-1 (500 ng). To analyze the precipitated proteins, Western blotting was performed using the 6X His tag antibody (Gentex, Cat# GTX115045-01, Zeeland, MI, USA) and anti-TIMP-1 (Abcam, Cat#ab61224).

### 2.4. Western Blot

Primary human monocytes were isolated the day before the experiment. On the next day, 1 × 10^6^ monocytes were starved for 2 h in RPMI 1640 media supplemented with 2% FBS and 1% P/S and stimulated with 12.5 ng/mL, 25 ng/mL, 50 ng/mL, 100 ng/mL, 200 ng/mL, or 400 ng/mL of TIMP-1 for 20 min at 37 °C in the same media. Subsequently, the media were removed and the samples lysed using LDS lysis buffer (ThermoFisher Scientific). The proteins were separated on a 12% SDS-Gel and plotted onto a PVDF membrane (GE Healthcare). As a marker, the CozyHi prestained protein ladder (highqu, Kraichtal, Germany) was used.

For antigen detection, the membranes were incubated with primary antibody (1:1000 in 5% BSA in TBS-T) overnight at 4 °C. On the next day, the membranes were washed and incubated with the secondary antibody (anti-rabbit HRP, 1:10,000, or anti-mouse HRP, 1:10000, in TBS-T for 1.5 h at RT). Membranes were developed with a chemiluminescent substrate (SuperSignal West Femto Maximum Sensitivity Substrate, ThermoFisher Scientific) and imaged using the Odyssey Fc Imaging System (LI-COR Biosciences, Lincoln, OR, USA) equipped with Image Studio software 1.51n.

### 2.5. Phospho-Kinase Array

The effect of TIMP-1 stimulation on the phosphorylation of kinases in THP-1 cells was conducted using a membrane-based human phospho-kinase antibody array kit (ARY003B, R&D Systems, Minneapolis, MN, USA) designed to profile 46 specific phosphorylation sites. Following the manufacturer’s instructions, THP-1 cells were stimulated with recombinant TIMP-1 (100 ng/mL) for the indicated time intervals. The corresponding cell lysates were incubated with the pre-blocked membrane overnight at 4 °C. On the second day, the membrane was extensively rinsed and incubated with biotinylated detection antibodies (antibody cocktail) at RT for 2 h. Thereafter, the membrane was exposed to a diluted streptavidin-horseradish peroxidase (HRP) solution at RT for 30 min, followed by visualization using a chemi-reagent mix and the Odyssey^®^ Fc imager (LI-COR Biosciences). The pixel density of the obtained spots was quantified using ImageJ software Version 1.51n and expressed as the relative phosphorylation level of the corresponding kinase.

### 2.6. Transwell Migration Assay

The chemotactic migration of primary human monocytes was analyzed using a Transwell-based assay as described previously [11]. Monocytes were isolated as mentioned above and cultured in RPMI 1640 medium with 10% FBS and 1% P/S overnight. Then, 1 × 10^6^ cells were transferred into 100 µL RPMI medium (2% FBS, 1% P/S) and were loaded into the upper chamber of the Transwell insert (5 µm, Corning, Kaiserslautern, Germany). Filters were placed into the wells (the bottom chamber) containing different concentrations of TIMP-1 (e.g., 50 ng/mL, 100 ng/mL, 200 ng/mL). The inhibitory effects of LN-2 (10 µg/mL), the IgG1 isotype control (10 µg/mL), and AMD3100 (10 µg/mL) on TIMP-1-mediated cell migration were determined by prior incubation of the inhibitors with cells at 37 °C for 1 h. The Transwell device was incubated overnight at 37 °C and 5% CO_2_. Cells that migrated into the bottom chamber were collected and counted by flow cytometry using CountBright™ absolute counting beads (Molecular Probes-Invitrogen, Karlsruhe, Germany).

### 2.7. Live Imaging in a 3D Chemotaxis Assay Setup

The 3D Chemotaxis µ-Slide device (ibidi GmbH, Munich, Germany) was used according to the manufacturer’s protocol. Briefly, primary human monocytes were isolated the day before the assay as previously described. Then, 5 × 10^6^ cells were seeded into a 1 mg/mL rat tail collagen type I matrix (Merck Millipore, Burlington, MA, USA) in DMEM medium. The 3D Chemotaxis µ-slides were incubated at 37 °C for 30 min and then exposed to different concentrations of TIMP-1 (i.e., 200 ng/mL, 400 ng/mL) or CCL2 (200 ng/mL). The inhibitory effects of LN-2 (20 µg/mL), the IgG1 isotype control (20 µg/mL), and AMD3100 (10 µg/mL) on TIMP-1-mediated 3D cell migration was determined by the addition of the inhibitor to the appropriate channel. Heat-inactivated TIMP-1 was incubated for 30 min at 95 °C (HI-TIMP-1) and served as a specificity control. Cell motility was tracked by time-lapse imaging every 2 min at 37 °C for 2 h using a Leica DMi8 microscope (Leica Microsystems, Wetzlar, Germany). Images were analyzed by manual tracking using ImageJ software Version 1.51n and the Chemotaxis and migration tool (ibidi GmbH).

### 2.8. Flow Adhesion Assay

The effect of TIMP-1 on monocyte adhesion under flow conditions to an endothelial monolayer was studied using a microfluidic chamber (μ-slide I 0.8, ibidi GmbH, Munich, Germany) and the ibidi Pump System (ibidi) as previously described [27]. Briefly, ~7 × 10^4^ HAoECs were seeded into the microfluidic chamber coated with collagen type I (ibidi, catalog #50201), the day before the experiment to obtain a confluent monolayer. MM6 cells were stained prior to the experiment with CFSE tracker (BioLegend). Cells were treated with different concentrations of TIMP-1 (e.g., 200 ng/mL, 400 ng/mL) with or without LN-2 (10 µg/mL) or IgG1 (10 µg/mL) at 37 °C for 2 h. MM6 cells were also treated with the same concentrations of TIMP-1 with or without inhibitors for 2 min, resuspended in assay buffer (1 × HBSS, 10 mM HEPES, 0.5% BSA) to a final concentration of 0.6 × 10^6^ cells/mL, and perfused through the flow chamber for 10 min at 37 °C with a laminar shear stress of 1.5 dyn/cm^2^ and 12.5 mbar. For the groups including LN-2 or IgG1, the MM6 and HAoEC cells were pretreated with the inhibitor or the isotype control for 1 h before the addition of TIMP-1. The adherent cells were then imaged using the Leica DMi8 microscope (10x magnification) and quantified using four randomly chosen areas of the microfluidic chamber. CFSE-positive cells adherent to the HAoEC monolayer were manually counted using Image J software Version 1.51n.

### 2.9. Proliferation Assay

Mouse vascular smooth muscle cells (VSMCs) were seeded at a density of ~3000 cells per well of a 96-well plate (Greiner, microclear, Kremsmünster, Austria) the day before the experiment. On the next day, the cells were stimulated with different concentrations of TIMP-1 as indicated with or without LN-2 (10 µg/mL) or the IgG1 isotype control (10 µg/mL). Cell proliferation was measured using an IncuCyte S3 device (Sartorius, Göttingen, Germany) located in a cell culture incubator (37 °C, 5% CO_2_). Images were acquired using a 10× objective every 3 h for 96 h. To assess the proliferation of the VSMCs, the images were analyzed using the IncuCyte software proliferation AI analysis tool v2020C. Of note, the mouse VSMCs were stimulated with human TIMP-1. Sequence alignment showed that both proteins were nearly identical. The data were analyzed using Matlab2022a. The raw data were fit using the logistic growth model.

### 2.10. Internalization Assay

THP-1 cells (1 × 10^6^ cells) were starved in RPMI1640 medium supplemented with 2% FBS and 1% P/S and were subsequently treated with different concentrations of recombinant TIMP-1 or MIF (as a control) at different time points under a 5% CO_2_ humidified atmosphere at 37 °C. After incubation, the internalization process was stopped by placing the cells on ice for 10 min. The cells were then washed twice with ice-cold PBS followed by flow cytometry buffer (PBS containing 0.5% BSA) and incubated for 1 h in the dark at 4 °C with the FITC anti-CD74 antibody, the APC anti-CXCR4 antibody, or the appropriate isotype control (IgG1). After incubation, the cells were washed thoroughly and analyzed using the BD FACSVerse™ (BD Biosciences, East Rutherford, NJ, USA). Quantification was performed using FlowJo software 1.51n.

### 2.11. Immunofluorescence Staining

THP-1 cells were seeded on glass coverslips in bottom chamber 4-well slides (ibidi) and incubated with poly-L-lysin (Sigma Aldrich, Deisenhofen, Germany) for 1 h. Then, the cells were fixed with 4% paraformaldehyde (PFA) (ThermoFisher Scientific) for 15 min, permeabilized with 0.1% Triton X-100 in PBS/BSA 1% blocking buffer (Cell Signaling Technology, Danvers, MA, USA) for 30 min at RT, and incubated overnight at 4 °C with primary antibodies against CD74 (AF7478) (R&D, Minneapolis, MN, USA) and TIMP-1 (ab1827) (Abcam, Cambridge, Great Britain). Non-specific isotype antibodies were used as negative controls. Species-specific Star488/Star635 secondary fluorescence antibodies (Abberior, Goettingen, Germany) were applied for 2 h at RT. The slides were embedded in Prolong^®^ Diamond antifade mountant (ThermoFisher Scientific) in the presence of 4′,6-diamidino-2-phenylindole (DAPI) to counterstain the nuclei. Digital images were acquired using a Leica DMi8 fluorescence microscope equipped with a digital camera (Leica Microsystems).

### 2.12. Flow Cytometry

The cell surface expression of CXCR4 and CD74 on THP-1 cells and the impact of TIMP-1 on the internalization of both receptors in primary human monocytes were analyzed by flow cytometry using APC-conjugated anti-CXCR4 (R&D) and FITC-conjugated anti-CD74 (R&D) or the corresponding APC- or FITC-conjugated isotype IgG controls. In brief, 1 × 10^6^ THP-1 cells or isolated primary human monocytes were first washed three times with ice-cold PBS supplemented with 0.5% BSA and then incubated with the above-mentioned antibodies against CD74 and CXCR4 or with the corresponding control IgGs for 1 h at 4 °C in the dark. After incubation, cells were washed thoroughly and analyzed using BD FACSVerse™ (BD Biosciences). Quantification was performed using FlowJo V10 software (Treestar).

### 2.13. Molecular Docking Analysis

The sequences were aligned using Clustal Omega. The superimposition of MIF (Pdb: 1MIF), MIF-2 (Pdb: 7MSE), and TIMP-1 (3V96) was performed using UCSF Chimera. The molecular interactions within the MIF–CD74, MIF-2–CD74, and TIMP-1–CD74 complexes were analyzed using HADDOCK (HADDOCK Webserver 2.4) [28,29]. The active residues were selected as previously described [24,30,31]. All complexes were finally modeled using UCSF Chimera. The data were analyzed using Matlab2022a.

### 2.14. Re-Analysis of scRNA-Seq Datasets

Human single-cell RNA seq datasets from patients undergoing carotid endarterectomy were re-analyzed using R and SeuratV4.2.0 (Alsaigh et al., 2022) or PlaqView using the “annotation as provided by the author” function (Zernecke et al., 2022, and Slenders et al., 2021) [32,33,34,35,36]. The dataset from Alsaigh et al. was previously published on genome expression omnibus (GSE159677) and re-analyzed in this study using R. For this study, we focused on the atherosclerotic core dataset. The data were analyzed according to the Seurat standard vignette. Briefly, the datasets of three patients were filtered (QC), merged, and corrected for batch effects using canonical correlation analysis (CCA). Doublets were removed using DoubletFinder [37]. The resolution was set to 0.58. Clusters were annotated according to the markers used by Alsaigh et al. including additional monocyte and macrophage markers to subcluster the monocyte/macrophage population.

### 2.15. Statistical Analysis

The statistical analysis was conducted using GraphPad Prism V8 software. The normality of the data was assessed prior to the analysis. Student’s *t*-test with Welch’s correction and one-way ANOVA were employed to perform the statistical analysis. To account for multiple comparisons, either Dunn’s post hoc, Tukey’s, or Sidak’s multiple comparisons tests were applied as appropriate. The results are presented as the mean ± the standard deviation (±SD). A *p*-value < 0.05 was considered statistically significant.

## 3. Results

### 3.1. Structural and Computational Insights into the Interaction between CD74 and TIMP-1

**Figure 1:**
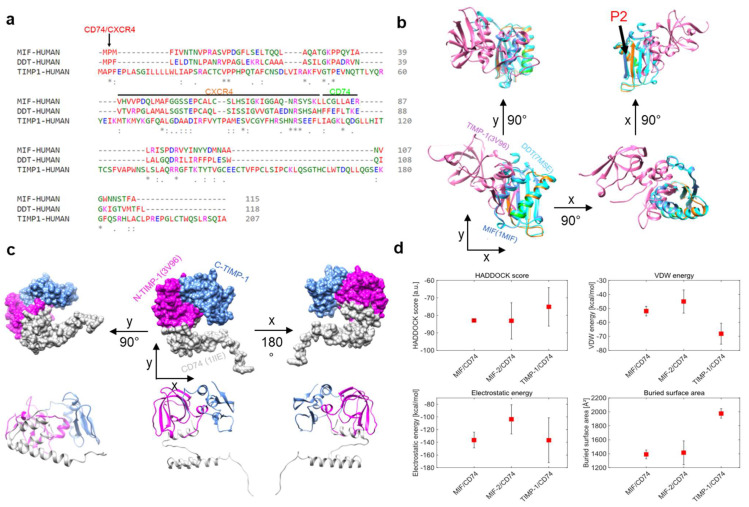
In silico analysis of TIMP-1–CD74 interaction and comparison of binding parameters between TIMP-1–CD74, MIF–CD74, and MIF-2–CD74 complexes. (**a**) Comparison/alignment of amino acid sequences of MIF (1MIF), MIF-2 (7MSE), and TIMP-1 (3V96). Potential sequence regions critical for receptor binding are marked by a line/arrow and color (CD74: green; CXCR4: orange; CD74/CXCR4: red). (**b**) Overlay of MIF, MIF-2, and TIMP-1 structures from different perspectives. The position of the conserved proline-2 is indicated (P2). (**c**) In silico protein–protein docking of TIMP-1 with a monomer of CD74 (1IlE, grey). The domains of TIMP-1 are color-coded (N-terminal domain, magenta; C-terminal domain, blue). Top panel, surface representation; bottom panel, ribbon representation. (**d**) Comparison of different docking parameters for the MIF–CD74, MIF-2–CD74, and TIMP-1–CD74 interactions. The average values were calculated using the 4 best structures of each cluster, and the error bars indicate the standard deviation of these values. * Conserved sequence (identical), : Conservative mutation, · Semi-conservative mutation.

MIF and D-DT/MIF-2 are established ligands of CD74 [13,38]. MIF also binds to CXCR4 [9], and recent evidence suggests that MIF-2 is an additional ligand for CXCR4 as well [30]. The recently suggested interaction between TIMP-1 and CD74 [22,24] calls for a structural comparison of the three ligands and their binding properties. We first performed multiple amino acid (aa) sequence alignments of MIF, MIF-2, and TIMP-1 (Figure 1a). Several clusters of residues were observed, in which the sequence of TIMP-1 contained identical residues or conservative or semi-conservative changes. Some of these clusters fall within the MIF sequence regions previously determined by experimental studies [39,40,41], to contribute to MIF receptor binding. For example, the proline-2 residue, which is evolutionarily conserved in the MIF protein family and was found to be important for the binding of MIF to CD74 and also CXCR4 [40,42], was also found in the TIMP-1 sequence in the same aligned position. Further similarities were noted in regions 49–55 and 72–78 (previously shown to be involved in CXCR4 binding of MIF), as well as 80–87 (relevant for CD74 binding) [39,40]. Experimental studies will need to clarify whether the corresponding residues in TIMP-1 are involved in MIF receptor binding or whether the sequence similarities are coincidental. Next, we performed in silico superimposing of the crystal structures of MIF (blue), MIF-2 (cyan), and TIMP-1 (magenta) to test for three-dimensional structural similarities (Figure 1b). The CD74/CXCR4 (red), CXCR4 (orange), and CD74 (green) binding sites were annotated in the superposition of the structures. The proline-2 residue was similarly positioned in MIF, MIF-2, and TIMP-1, whereas the CXCR4 and CD74 binding sites of MIF (and MIF-2) did not overlay the respective structural region in TIMP-1. However, a closer look at the 3D structure revealed that TIMP-1 harbors two β-sheets and an α-helix that resemble the CXCR4 binding site (ß-sheet and α-helix) and the CD74 binding site (aa 80–87 in α-helix), but seem to be spatially shifted in the performed overlay.

In order to gain further insight into the interaction between TIMP-1 and CD74 compared to the MIF–CD74 and MIF-2–CD74 interactions, in silico protein–protein docking was performed. For this purpose, we used the HADDOCK 2.4 webserver [12,13]. Our analysis employed TIMP-1 (aa 1–178) and a monomer of CD74 (aa 118–192, extracellular part) and suggested that TIMP-1 binds CD74 through its N-terminal region (Figure 1c). This approach also aimed to explore different docking parameters and energies associated with the TIMP-1–CD74 interaction and compared them with those observed for the MIF–CD74 and the MIF-2–CD74 interactions in order to understand the similarities and differences between these interactions (Figure 1d). We analyzed four different parameters: the HADDOCK score, the Van der Waals (VdW) energies, the electrostatic energies, and the buried surface area of the dockings. The results indicated that the HADDOCK score of the MIF–CD74 interaction (−82.9 a.u.) is highly comparable to that of the MIF-2–CD74 interaction (−83.1 a.u.), while the binding of TIMP-1 to CD74 appeared to be weaker (−75.1 a.u.). Of note, the VdW energies were more pronounced in the interaction between TIMP-1 and CD74 than in the interaction between MIF (−2) and CD74. Interestingly, MIF and TIMP-1 showed comparable electrostatic energies, which are crucial for binding, while this parameter was lower for MIF-2. Moreover, the buried surface area of MIF and MIF-2 was quite similar and considerably smaller than the one of the TIMP-1–CD74 interface.

In summary, our in silico analysis revealed that TIMP-1 shares several residues with MIF and MIF-2 that are important for binding to CD74 or CXCR4. The superimposition of the MIF/MIF-2/TIMP-1 tertiary structures indicated that the structures are different at first sight, but a closer inspection of the regions important for receptor binding showed distinct similarities. The protein–protein docking analysis further confirmed the critical role of the N-terminus of TIMP-1 in its interaction with CD74, as previously suggested [24]. The ana-lysis of different docking parameters suggested that, while the MIF–CD74 and the MIF-2–CD74 interactions are similar, the TIMP-1–CD74 interaction is comparatively weaker. In addition, the buried surface area of the MIF–CD74 and the MIF-2–CD74 is almost identical, whereas the TIMP-1–CD74 interface is considerably larger.

### 3.2. TIMP-1 Interacts with Membrane CD74 and Its Soluble Ectodomain Fragment sCD74

**Figure 2:**
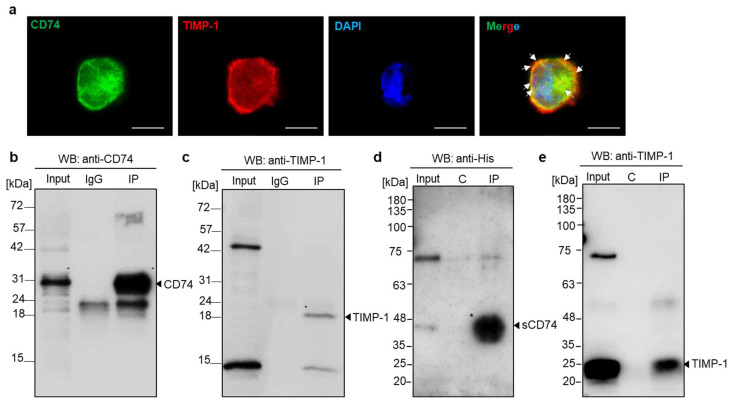
Validation of the interaction between TIMP-1 and CD74 by immunocytochemistry and immunoprecipitation analysis. (**a**) Colocalization of TIMP-1 and CD74 in THP-1 monocytes. Fluorescence microscopy analysis costained with anti-TIMP-1/Star635 secondary antibody (red), anti-CD74/Star488 secondary antibody (green), and DAPI (blue, DNA labeling in nuclei). The merged image shows the overlay (yellow) of the two channels demonstrating TIMP-1 colocalization with CD74. Arrows indicate membrane regions of substantial colocalization. Scale bar: 10 µm. (**b**,**c**) Immunoprecipitation of TIMP-1 with cell surface CD74. Semi-endogenous pull-down assay using protein cell lysate from HEK29 cells transfected with FLAG-tagged CD74 and recombinant TIMP-1 analyzed by Western blotting with anti-CD74 (**b**) and anti-TIMP-1 (**c**) antibodies. (**d**,**e**) TIMP-1 interacts with the soluble CD74 ectodomain fragment (sCD74). Immunoprecipitation of His-tagged sCD74 and recombinant TIMP-1 and the analysis of precipitated proteins by Western blotting using anti-His (**d**) and anti-TIMP-1 (**e**) antibodies. C: control.

For the experimental examination of the molecular interaction between TIMP-1 and CD74 and to test whether TIMP-1 binding to CD74 also occurs in monocytes, we evaluated the subcellular colocalization of TIMP-1 and CD74 in monocytic THP-1 cells, which express CD74 (Supplementary Figure S1) and TIMP-1 [26]. Immunocytochemistry analysis demonstrated colocalization of endogenous TIMP-1 and CD74 with a predominant presence on the cell surface (Figure 2a). Subsequently, we verified the interaction through co-immunoprecipitation using whole-cell lysates of HEK293 cells that had been transfected with N-terminal FLAG-tagged human CD74. The semi-endogenous pulldown of proteins from cell lysates and recombinant human TIMP-1 was carried out with anti-FLAG antibodies and magnetic G beads. Western blotting using anti-human CD74 or anti-human TIMP-1 antibodies demonstrated successful pulldown and a specific band for TIMP-1, which was absent when the pulldown was performed without recombinant TIMP-1 preincubation (Figure 2b,c).

The 37 kDa form of the cell-surface-expressed CD74 membrane protein may be subject to ectodomain shedding to produce a 25 kDa soluble CD74 ectodomain fragment (sCD74). Shedding has, for example, been observed for liver cells, and sCD74 has been shown to bind to MIF [39]. Based on our results validating the binding of TIMP-1 to full-length cell-surface-expressed CD74, we next wished to investigate whether TIMP-1 could also form a complex with sCD74. To this end, we conducted immunoprecipitation of His-tagged sCD74 and recombinant human TIMP-1. As illustrated in Figure 2d,e, TIMP-1 was found to co-precipitate with sCD74, while no interaction was observed in a control pulldown without anti-His antibody. These findings support the hypothesis that the extracellular, N-terminal domain of CD74 is necessary and sufficient for binding to TIMP-1.

### 3.3. TIMP-1 Promotes the Internalization of CD74, but Not CXCR4

In order to further investigate the interaction between TIMP-1 and CD74, we next asked whether TIMP-1 affects the cell surface expression of CD74 by promoting its internalization. At the same time, receptor internalization assays are a valuable method to confirm functional ligand/receptor binding events.

**Figure 3:**
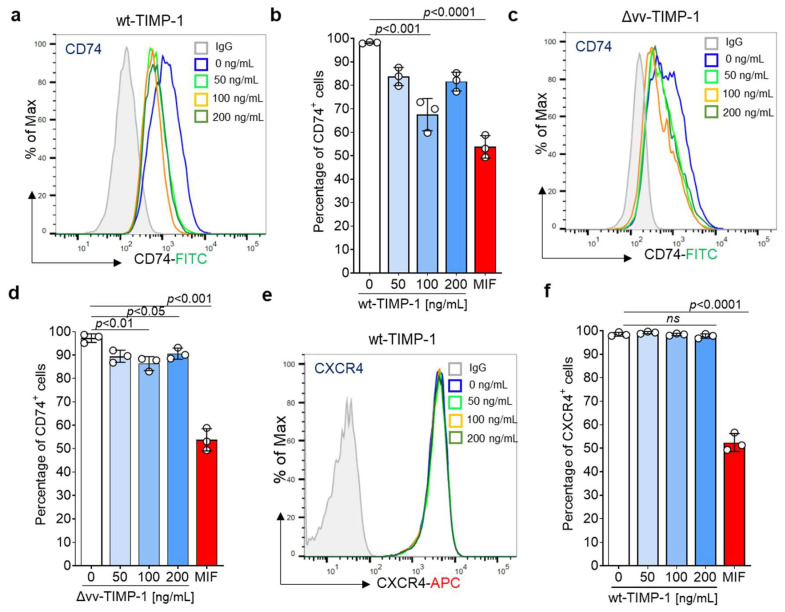
Impact of TIMP-1 and a non-MMP inhibitory mutant (Δvv-TIMP-1) on the internalization of cell surface CD74 and CXCR4 in THP-1 cells. CD74 and CXCR4 cell surface expression were monitored using FITC-conjugated anti-CD74 and APC-labeled anti-CXCR4 antibodies, respectively. Full-length TIMP-1 (wtTIMP-1; (**a**,**b**)) promotes the internalization of CD74 in THP-1 cells in a dose-dependent manner (minimum at 100 ng/mL). Δvv-TIMP-1 (**c**,**d**) also enhances the internalization of CD74, but the effect is smaller and lacks a dose behavior. (**a**,**c**) Representative histograms. (**b**,**d**) Quantification of CD74 cell surface expression from three independent experiments. MIF (200 ng/mL) was used as a positive control. (**e**,**f**) Stimulation of THP-1 with TIMP-1 showed no effect on CXCR4 internalization. (**e**) Representative histogram, (**f**) Quantification of CXCR4 cell surface expression from three independent experiments. Data are means ± SD.

We conducted an internalization assay using THP-1 cells, which express both MIF receptors CD74 and CXCR4 (Supplementary Figure S1). Cells were starved and then treated with varying concentrations of full-length TIMP-1 (wt-TIMP-1), the non-MMP inhibitory variant (Δvv-TIMP-1) mutant, or MIF (used as the positive control). Cell surface CD74 was monitored by flow cytometry. Confirming our in silico docking, colocalization, and co-immunoprecipitation data, cell stimulation with wt-TIMP-1 significantly elicited CD74 internalization in a dose-dependent manner, similar to the effects of MIF, with an optimal concentration of 100 ng/mL (Figure 3a,b). The Δvv-TIMP-1 mutant had a moderate, albeit still significant effect, on mediating CD74 internalization, but did not show a dose-optimum behavior (Figure 3c,d). This may indicate that TIMP-1-mediated CD74 internalization is independent of MMP activity and that the mutated residues partially contribute to the CD74 binding interface.

MIF and its homolog MIF-2 are ligands of CXCR4 and induce the internalization of this receptor [9,30]. Based on the multiple-sequence alignment combined with the superimposition of the crystal structures, which showed the presence of shared stretches of amino acids crucial for the CXCR4 interaction, we considered that TIMP-1 may also bind to CXCR4. To test this hypothesis, we attempted to determine if TIMP-1 enhances CXCR4 internalization in the same cell line. However, the incubation of THP-1 cells with TIMP-1 failed to induce CXCR4 internalization, suggesting that TIMP-1, unlike MIF, does not bind to CXCR4 and/or does not support its internalization (Figure 3e,f).

In summary, our results indicated that TIMP-1 directly binds to CD74, but not to CXCR4, to induce its internalization in a dose-dependent manner.

### 3.4. TIMP-1 Triggers Downstream Signaling Pathways in THP-1 Cells

**Figure 4:**
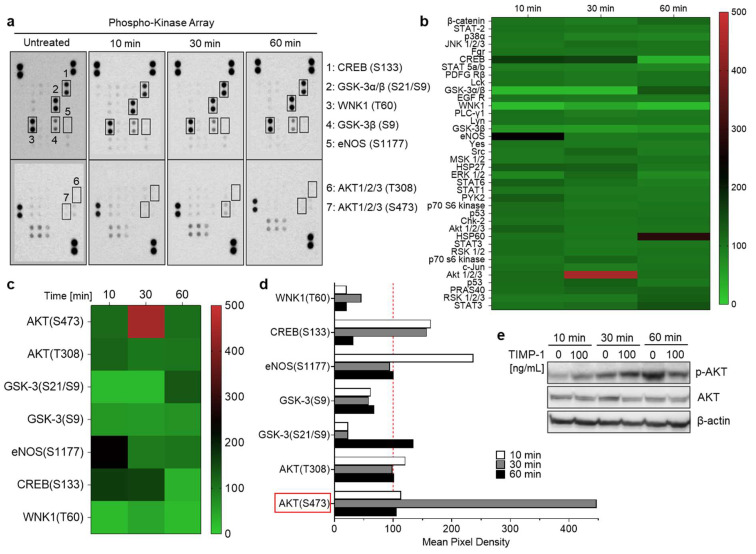
TIMP-1 promotes the activation of serine/threonine kinases in THP-1 monocytic cells. Cells were incubated under serum-free conditions in the absence (untreated or 0) and presence of recombinant TIMP-1 (100 ng/mL) for different time intervals, as indicated. (**a**) Total-protein cell lysates were obtained and analyzed using a membrane-based phosphokinase array, detecting 46 different kinase phosphorylation sites (seven of the phosphorylated substrates are indicated; the respective phosphorylated amino acids in brackets). (**b**) Densitometric quantification of the spots in (**a**) and representation as a heatmap. The quantification is based on mean values from duplicate determinations in percent to untreated control cells (100%). The heatmap shown provides an overview of the changes in phosphorylation of all 46 measured kinase substrates/sites at all three determined time points. (**c**) Selection of the heatmap in (**b**) with a focus on those substrates/sites with the most-substantial alterations. (**d**) Representation of the densitometric quantification according to the selection of substrates/sites depicted in (**c**) as a bar diagram. The dotted red line indicates the baseline value of the control. (**e**) Western blot analysis of cell lysates using antibodies against phosphorylated AKT (p-AKT), total Akt (AKT), and β-actin as the loading control.

Given that TIMP-1 interacts with CD74 to promote its internalization, we next aimed to investigate whether TIMP-1 induces downstream signals in THP-1 cells. To test this hypothesis, cells were incubated without and with recombinant TIMP-1 for different time intervals. Changes in the phosphorylation of kinases were determined using an antibody array designed to profile 46 specific phosphorylation sites (Supplementary Table S1). The exposure of THP-1 cells to TIMP-1 evoked significant alterations in the levels of the phosphorylation of the cAMP response element-binding protein (CREB), glycogen synthase kinase-3 (GSK-3) α and β, lysine-deficient protein kinase 1 (WNK1), endothelial nitric oxide synthase (eNOS), and protein kinase B (AKT1/2/3) (Figure 4a–d).

In response to TIMP-1, phosphorylation levels were most-clearly augmented in CREB, eNOS, and AKT, but diminished in WNK1 and GSK3β (Figure 4b–d). Of note, massive phosphorylation of AKT occurred at serine residue 473 (S473) after 30 min of incubation with TIMP-1, while threonine 308 (T308) was not affected (Figure 4c,d). These findings suggest that TIMP-1 promotes the activation of AKT-dependent signaling pathways through specific phosphorylation of AKT’s serine residue 473 in monocytic THP-1 cells (Figure 4a–d). This was confirmed by Western blot analysis, demonstrating increased AKT phosphorylation after 30 min of incubation with TIMP-1 with a decrease after 60 min compared to untreated control cells (Figure 4e).

Taken together, these findings indicated that TIMP-1 modulates the activity of various serine/threonine kinases, most prominently AKT, in monocytic cells, confirming the cytokine-like function of TIMP-1.

### 3.5. TIMP-1 Induces Migration of Primary Human Monocytes through CD74, but Not through CXCR4

**Figure 5:**
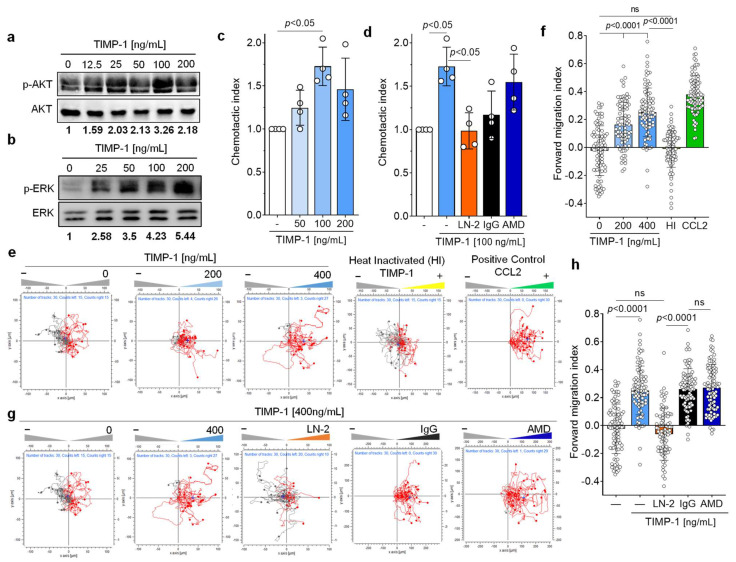
TIMP-1 triggers the chemotactic migration of primary human monocytes via CD74. (**a**,**b**) Recombinant TIMP-1 induces phosphorylation of AKT (**a**) and ERK1/2 (**b**) in primary human monocytes in a dose-dependent manner. Cell lysates from isolated human monocytes stimulated with different concentrations of TIMP-1 were analyzed by Western blot using anti-pAKT, anti-AKT, anti-pERK1/2, and anti-ERK1/2 antibodies, as indicated. (**c**) Dose-dependent Chemotaxis of human primary human monocytes in response to TIMP-1 as determined by the Transwell migration assay (n = 4 biological replicates). (**d**) The Transwell assay showed the involvement of CD74, but not CXCR4, in the TIMP-1-mediated Chemotaxis of monocytes. The blocking anti-CD74 antibody (LN2) and the CXCR4 antagonist (AMD3100) were used to test the causal role of both receptors in TIMP-1-triggered Chemotaxis (n = 4 biological replicates). (**e**) TIMP-1 triggers the 3D Chemotaxis of human monocytes in a dose-dependent manner (red traces). The unstimulated control (gray traces) reflects random motility processes. A representative experiment is shown. Migration trajectories of 30 randomly selected cells per treatment group were recorded. Heat-inactivated (HI) TIMP-1 and CCL2 were used as negative and positive controls, respectively. (**f**) Quantification of (**e**) using the forward migration index (FMI) as the Chemotaxis activation parameter. Shown are the migration trajectories of one experiment out of n = 3 independent experiments performed. (**g**) Involvement of CD74, but not CXCR4, in TIMP-1-triggered migration, as determined by 3D real-time Chemotaxis of human monocytes. (**h**) Quantification of (**g**) using the FMI as the Chemotaxis activation parameter. Shown are the migration trajectories of one experiment out of n = 3 independent experiments performed. Migration trajectories of 30 randomly selected cells per treatment group were recorded. Data are means ± SD.

The cytokine-like properties of TIMP-1, in particular its activating effect on the PI3K/AKT pathway, next led us to investigate the importance of TIMP-1-mediated signals in primary human monocytes. TIMP-1 elicited a dose-dependent phosphorylation of AKT with an optimal concentration of 100 ng/mL (Figure 5a). Moreover, TIMP-1 stimulated ERK1/2 phosphorylation, a downstream signal of the MAPK pathway (Figure 5b).

As both pathways are prominently involved in the regulation of cell migration [43], we subsequently assessed the migratory potential of primary human monocytes upon exposure to TIMP-1 using a Transwell-based Chemotaxis assay. The results, depicted in Figure 5c, demonstrated a dose-dependent chemotactic migration of monocytes in response to TIMP-1, displaying a bell-shaped dose–response curve. Notably, the peak concentration of TIMP-1-induced Chemotaxis was also found to be at 100 ng/mL, consistent with the concentration previously determined to be optimal for AKT- and ERK1/2 phosphorylation (Figure 5a,b), and the degree of TIMP-1-mediated chemotactic activation was similar to the chemotactic indexes previously determined for MIF or MIF-2. We next tested the impact of a neutralizing antibody against CD74 (anti-CD74, LN-2) and an isotype control immunoglobulin (IgG1) on TIMP-1-triggered Chemotaxis to verify the requirement of CD74 in this process. Remarkably, the blockade of CD74 abolished TIMP-1-triggered migration compared with the control (IgG1) (Figure 5d). Of note, co-incubation of monocytes with the CXCR4-specific small molecule inhibitor plerixafor/AMD3100 (AMD) showed no effect on their migration toward TIMP-1 (Figure 5d). These results suggest that TIMP-1 promotes monocyte Chemotaxis through CD74, but not CXCR4.

To provide further evidence for the effects of TIMP-1 on monocyte migration, we applied a 3D-Chemotaxis-based assay. Primary monocytes were subjected to a chemotactic gradient of TIMP-1 and the trajectories of individual cells analyzed by in vivo time-lapse imaging. We first determined the optimal concentration at which TIMP-1 affects monocyte migration/motility and found that a significant response was observed at both 200 and 400 ng/mL of TIMP-1 compared with the control (Figure 5e,f). The effect size of the 400 ng/mL TIMP-1 dose was similar to that of CCL2 (positive control), resulting in a shift of the center of mass (+) to the TIMP-1 gradient (right reservoir) (Figure 5e) and an increased forward migration index (FMI: 0.25) compared to 200 ng/mL (FMI: 0.16) (Figure 5f). Moreover, heat-inactivated (HI) TIMP-1 showed no effect on monocyte Chemotaxis (FMI: −0.01), suggesting its specific chemotactic activity. We also examined the involvement of CD74 and CXCR4 in the TIMP-1-induced 3D Chemotaxis of monocytes by blocking both receptors with anti-CD74 (LN2) and AMD3100, respectively. The results clearly showed that LN-2 completely abolished the migratory effect induced by TIMP-1 (FMI: −0.06), while the IgG1 control immunoglobin did not affect the migratory behavior of monocytes under the same conditions (FMI: 0.26) (Figure 5g,h). In contrast, the inhibition of CXCR4 by AMD3100 did not lead to a reduction in the chemotactic response (FMI: 0.27) (Figure 5g,h), suggesting that CXCR4 is not involved in TIMP-1-mediated monocyte migration.

In summary, our results demonstrated that TIMP-1 potently promotes the chemotactic migration of monocytes and suggested that it exerts its cytokine/chemokine-like activities through CD74, but not through CXCR4 to trigger the migration of monocytes.

### 3.6. TIMP-1 Facilitates the Adhesion of Human Monocytes on Endothelial Monolayers through CD74

The involvement of the TIMP-1–CD74 axis in monocyte migration implies its potential role in monocyte recruitment during inflammatory processes. Monocyte recruitment and adhesion are closely interlinked and known as one of the main mechanisms of action during inflammation [9,27]. In line with this, previous studies have demonstrated that MIF triggers both the migration and adhesion of monocytes, mediated by CXCR4, but also with the involvement of CD74 [9,44].

**Figure 6:**
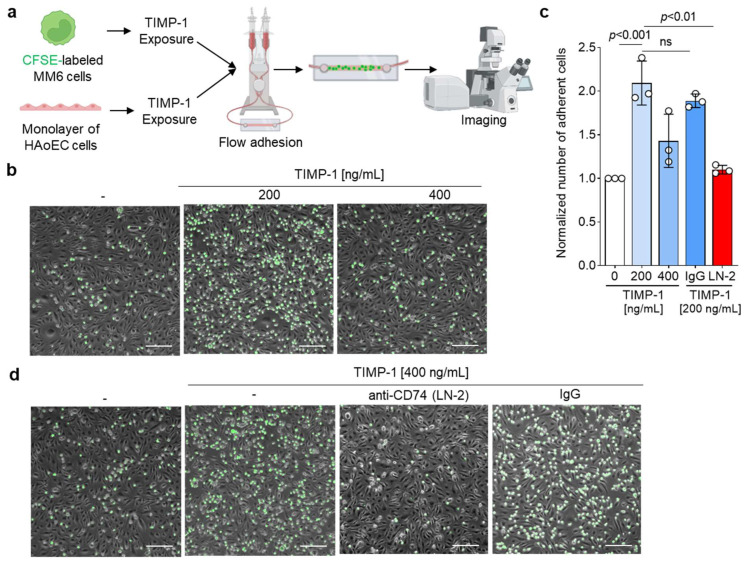
TIMP-1 induces the adhesion of MonoMac6 cells to monolayers of human aortic endothelial cells (HAoEC) under flow conditions. (**a**) Schematic to illustrate the experimental procedure of the flow adhesion assay. (**b**) Representative images of the dose-dependent effect of TIMP-1 on MonoMac6 arrest of HAoECs. Treatment with TIMP-1 as indicated in (a). (**c**) Representative images testing the involvement of CD74. Prior to flow adhesion, CFSE-stained MonoMac6 and HAoEC cells were pretreated with 10 µg/mL anti-CD74 (LN2) or the IgG1 isotype control for 1 h. (**d**) Quantification of the normalized number of adherent MonoMac6 cells and statistical analysis from the images recorded according to (**b**,**d**) (n = 3 biological replicates each). Adherent cells were counted manually using ImageJ. Data are means ± SD. The cartoon was created with biorender.com (accessed on 15 January 2023).

To determine if TIMP-1 also enhances monocyte adhesion, we conducted a flow cell adhesion assay, in which MonoMac6 monocytes were perfused over a confluent monolayer of human aortic endothelial cells (HAoECs) under flow stress conditions (Figure 6a). Microscope images of the cells adhered on the monolayer (Figure 6b) demonstrated that TIMP-1 promotes the arrest of MonoMac6 cells on HAoECs in a dose-dependent manner, with an optimal concentration of 200 ng/mL and a significant two-fold increase of adherent cells compared to the control (Figure 6d).

We also verified the involvement of CD74 in TIMP-1-mediated monocyte adhesion by preincubation of cells with the blocking antibody LN2. The blockade of CD74 significantly inhibited cell arrest and showed a comparable effect to the negative control (Figure 6c,d). Isotype control immunoglobulin had no effect on monocyte adhesion triggered by TIMP-1 (Figure 6c,d). Taken together, our data showed that TIMP-1 not only promotes monocyte migration, but also monocyte adhesion through CD74.

### 3.7. TIMP-1 Engages CD74 to Induce Growth and Proliferation of VSMCs

**Figure 7:**
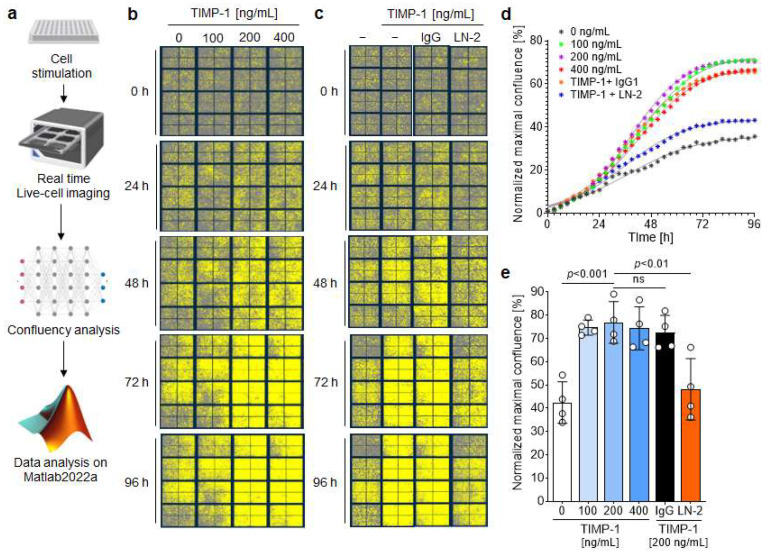
TIMP-1 promotes the proliferation of vascular smooth muscle cells (VSMCs) through CD74. (**a**) Flow chart illustrating the real-time proliferation assay and the analysis flow. VSMCs were treated with different concentrations of TIMP-1 for the indicated time interval and proliferation monitored using the IncuCyte Live Cell Imaging System. (**b**) Representative images show the dose- and time-dependent effect of TIMP-1 on the proliferation of VSMCs. Cells are highlighted in yellow. (**c**) Representative images showing the involvement of CD74 in the time- and dose-dependent TIMP-1-mediated VSMC proliferation. Cells were treated with 10 µg/mL anti-CD74 (LN2) or the IgG1 isotype control. (**d**) Confluence curves showing the proliferation of VSMCs. Phase object confluence was determined by the artificial intelligence (AI) of the IncuCyte software. For comparison purposes, the base confluence was normalized to 1. Data points were fit using the logistic growth model. (**e**) Quantification and statistical analysis of VSMC proliferation induced by TIMP-1–CD74. Normalized maximal confluence of the different treatment groups according to (**d**) was compared (n = 4 biological replicates each). Data are means ± SD. The scheme was created with biorender.com.

As demonstrated previously, the TIMP-1–CD74 axis promotes the activation of the PI3K/AKT pathway to promote monocyte migration and adhesion. This pathway also plays an important role in cell proliferation [45]. Interestingly, TIMP-1 has been shown to stimulate proliferation of human aortic smooth muscle cells, an effect then attributed to its MMP inhibitory activity [46]. We investigated the potential proliferative effect of TIMP-1 on mouse VSMCs and performed real-time concentration–response studies using the IncuCyte Live Cell Imaging System (Figure 7a). Human VSMCs were stimulated with various concentrations of TIMP-1, and the cell confluency was monitored in real-time by capturing four images every three hours for 96 h.

Figure 7b shows representative images demonstrating a dose-dependent effect of TIMP-1 on cell growth and proliferation. Significant effects were observed at an optimal concentration of 200 ng/mL TIMP-1 (Figure 7b,d,e). Moreover, there was a pronounced increase in cell growth between 24 and 72 h compared to untreated cells for all three tested TIMP-1 concentrations, as shown by the kinetics (Figure 7d). After 72 h, the proliferation rate reached a plateau (Figure 7d). Subsequently, the involvement of CD74 in TIMP-1-mediated VSMCs’ proliferation was examined in the presence of the anti-CD74 antibody LN2 or its IgG1 control. The blockade of CD74 led to a significant attenuation of cell growth comparable to the untreated control group (Figure 7c–e). On the contrary, the IgG1 control did not affect TIMP-1-elicited VSMC cell proliferation (Figure 7d,e).

All in all, our results showed that TIMP-1 dose-dependently promotes the proliferation of VSMCs through its receptor CD74.

### 3.8. The TIMP-1–CD74 Axis in Human Atherosclerotic Lesions as Re-Analyzed from scRNA-Seq Datasets

The observed impact of the TIMP-1–CD74 axis on monocyte migration, adhesion, and VSMC proliferation suggested that this axis may also play a role in inflammatory processes driving the progression of atherosclerosis. To test this hypothesis, we determined the expression patterns of TIMP-1 and CD74 in human atherosclerotic lesions, by re-analyzing an available single-cell RNA sequencing (scRNA-seq) dataset (NCBI GEO; GSE159677) obtained from aortic core tissue specimens of atherosclerotic patients undergoing carotid endarterectomy (CEA) (Alsaigh et al. [34]).

**Figure 8:**
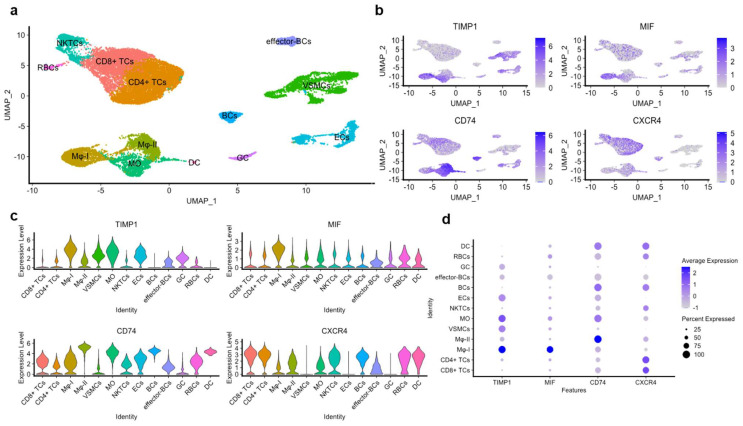
Re-analysis of an scRNA-seq dataset obtained from the atherosclerotic core of atherosclerotic patients undergoing carotid endarterectomy (CEA). The scRNA-seq dataset analyzed was from the NCBI GEO (National Center for Biotechnology Information Gene Expression Omnibus; NCBI GEO; GSE159677) and is based on material from three human CEA patients (Alsaigh et al. [34]). Here, re-analysis was conducted for the expression of TIMP-1, CD74, MIF, and CXCR4 in the different single-cell populations. (**a**) Uniform Manifold Approximation and Projection (UMAP) of the atherosclerotic core (AC) with thirteen clusters identified by Louvain clustering. (**b**) Feature plots, (**c**) violin plots, and (**d**) dot plots of the genes of interest in the different cell populations found in the AC including B cells (BCs); T cells (TCs); natural killer T cells (NKTCs); red blood cells (RBCs); vascular smooth muscle cells (VSMCs); endothelial cells (ECs); granulocytes (GCs); dendritic cells (DCs); monocytes (MOs); and macrophages (MΦ).

Employing Louvain clustering, we were able to discern thirteen distinct atherogenic cell populations, comprising T cells (CD8+ and CD4+), B cells, VSMCs, endothelial cells (ECs), dendritic cells (DCs), granulocytes, macrophages (MΦ), and monocytes (Figure 8a). The examination of the TIMP-1 expression profile in these cell populations demonstrated a marked and elevated expression in VSMCs, ECs, monocytes, and MΦ. A similar expression pattern was determined for CD74, with only minor variations in B cells and VSMCs, as evidenced by the Uniform Manifold Approximation and Projection (UMAP) and the violin plots (Figure 8b,c). Additionally, the expression level of TIMP-1 was comparable to that of MIF, but not to that of CXCR4 in the same populations (Figure 8c,d).

Interestingly, we observed two distinct macrophage (MΦ) populations, identified by the high expression of specific marker genes in each cell group (Figure 8a). These were classified as MΦ-I (characterized by elevated expression of *Spp1*, *Fabp5*, and *Cstb*) and MΦ-II (marked by elevated expression of *C1qa*, *Ms4a6*, and *Hla-Dqa1*). Of note, MΦ-I macrophages expressed high levels of TIMP-1 comparable to MIF, while exhibiting minimal expression of both CD74 and CXCR4 (Figure 8c,d). Conversely, MΦ-II macrophages displayed less-detectable expression of TIMP-1 or MIF, but instead, a high expression of CD74, suggesting differential roles of TIMP-1 and CD74 in these populations.

In order to corroborate the expression pattern identified by the analysis of the scRNA-seq dataset by Alsaigh et al. [34], we conducted a comparative analysis of our findings using datasets from Zernecke et al. [36] and Slenders et al. [35], who also studied human atherogenic lesions in CEA specimens at the single cell level. Remarkably, consistent findings were obtained, further supporting the high expression of both TIMP-1 and CD74, as well as MIF, in the MO, MΦ, and VSMC populations (Table 1). This observation underscores the potential involvement of the TIMP-1–CD74 axis in atherogenesis. Moreover, the somewhat distinct expression profile of TIMP-1 versus CD74 in macrophages and VSMCs was also observed in these datasets (Supplementary Figures S2 and S3).

Taken together, these findings support the notion that TIMP-1, serving as a novel ligand for CD74, may exhibit proatherogenic effects, which may result from its ability to regulate monocyte recruitment and VSMC proliferation via CD74.

## 4. Discussion

Previously, Schoeps et al. [24] and Hoeberg et al. [22] showed that TIMP-1 interacts with CD74. In the present study, we performed a sequence alignment analysis between the known CD74 ligands MIF and D-DT/MIF-2 and found that TIMP-1 shares key residues with both proteins that were previously determined to be important for MIF (MIF-2) binding to CD74 and/or CXCR4. However, the superposition of the tertiary structures of all three CD74 ligands and the analysis of various docking parameters indicated closer similarities for the CD74 interactions of MIF and MIF-2 CD74 compared to the interaction between TIMP-1 and CD74, which appears to be weaker. Consistent with one of the previous studies [24], our in silico protein–protein docking confirmed the critical role of the N-terminus of TIMP-1 for CD74 binding. The interaction was further confirmed by colocalization and immunoprecipitation studies in THP-1 monocytic cells and in primary human monocytes. Additionally, a direct interaction between TIMP-1 and CD74 was confirmed by a CD74 receptor internalization assay demonstrating a TIMP-1 concentration-dependent effect on the cell surface levels of CD74, but not CXCR4. Together, our docking and biochemical data clearly confirmed the previously suggested role of TIMP-1 as a novel ligand for the MIF receptor CD74 and extend the previous findings by characterizing the CD74 binding properties of TIMP-1 in comparison with those of MIF and MIF-2. Moreover, the binding data (as well as functional data; see the discussion below) suggested that TIMP-1 does not bind to the MIF receptor CXCR4.

TIMP-1 has been reported to activate both the PI3K/AKT and MAPK signaling pathways via its metalloprotease inhibitor function in the context of breast and colon cancer [47,48]. Besides this activity, TIMP-1 has also been shown to interact with the integrin/CD63 complex on the cell surface to activate the PI3K signaling pathway [49]. In line with this, we showed that TIMP-1-mediated activation of both signaling pathways is not restricted to cancer cells, but also expands to immune cells, particularly primary monocytes. CD74 has also been shown to facilitate MAPK and PI3K/AKT signaling following stimulation with MIF to regulate leukocyte recruitment and enhance inflammation [11,13,44,50,51]. Applying a Transwell migration assay and 3D-based real-time Chemotaxis, our data suggested that TIMP-1 is a novel chemoattractant for primary monocytes that triggers migration through CD74 in a dose-dependent fashion. To the best of our knowledge, this study is the first to show the chemotactic activity of TIMP-1, indicating that its cytokine/chemokine-like properties may potentially contribute to leukocytes trafficking, specifically monocytes, during inflammatory processes. As the TIMP-1 structure lacks the classifying motifs of *bona fide* chemokines such as the signature N-terminal cysteine motif or the chemokine fold, we would propose to designate TIMP-1 as a novel atypical chemokine (ACK), similar to other ACKs such as MIF [52,53]. Consistent with this notion, Olafsson et al. [54] showed that, under inflammatory conditions, DCs exhibit high secretion of TIMP-1, which in turn enhances the hypermotility of these cells through the involvement of the β-integrin1/CD63 complex.

An interesting finding of our study is that TIMP-1 also interacts with the soluble CD74 ectodomain fragment sCD74, which has been reported to regulate the activities of MIF [39,55]. It should be noted that sCD74 interacts with circulating MIF and attenuates MIF/CXCR4-mediated AKT phosphorylation [55]. Similarly, Fukuda et al. recently demonstrated that THP-1 cells exposed to IFN-γ upregulate the secretion of sCD74, which suppresses cell growth by inhibiting the MIF–CD74/AKT survival pathway [56]. Whether sCD74 also negatively regulates TIMP-1-mediated effects remains unclear and requires further investigation.

In addition to its chemotactic activity, our results indicated that TIMP-1 exerts another cytokine/chemokine-like property by dose-dependently modulating the adhesion of primary monocytes to HAoECs monolayers. This activity was also abrogated by the blockade of CD74. In contrast, Mostafa Mtairag et al. [57] showed that IL-10 regulates the transcriptional levels of TIMP-1 in MonoMac6 cells, which, in turn, significantly reduced their adhesion to HUVEC monolayers. However, this inhibitory effect was observed in the presence of IL-10 and/or appeared to be dependent on the MMP-9 inhibitory activity of TIMP-1 [57]. Here, we investigated the direct effect of TIMP-1 of the adhesion of human primary monocytes, providing evidence that these effects are driven by its chemokine-like activity engaging CD74. As non-MMP-blocking variant Δvv-TIMP-1 only partially promoted the internalization of CD74 compared to wildtype TIMP-1, it is conceivable that TIMP-1-induced monocyte adhesion is independent of its MMP inhibitory activity. Interestingly, the involvement of the TIMP-1–CD74 axis in both monocyte migration and adhesion suggested that TIMP-1, similar to MIF, may exhibit proatherogenic properties.

In addition to monocytes, VSMCs are well-known key regulators of the pathogenesis of atherosclerosis [58,59]. In one previous study, TIMP-1 was reported to stimulate the proliferation of human aortic smooth muscle cells (AoSMCs) through the activation of the MAPK and PI3K signaling pathways [46]. In accordance with these findings, we found that TIMP-1 activates CD74 to stimulate the proliferation of VSMCs in a concentration-dependent manner. An antibody neutralization approach clearly implicated CD74 as the TIMP-1-driven receptor in this process, as antibody blockade reduced the proliferation rate back to baseline values.

CD74 was demonstrated to play a significant role in inflammatory processes, mostly via its effects of inflammatory cell survival and proliferation [12,60]. Despite its importance, CD74 lacks an intracellular signal-transducing domain due to its short cytoplasmic tail. Consequently, signaling mediated by MIF–CD74 necessitates the presence of an accessory molecule acting as a signal-regulated co-receptor [60]. To this end, CD44 has been found to promote B cell survival and proliferation through the activation of PI3K/AKT signaling and anti-apoptotic protein expression of Bcl2 upon the activation of CD74 [50,61]. In addition to CD44, CD74 can also form a heteromeric complex with the chemokine CXCR4 to promote cell migration [11,62,63]. The evidence obtained in our current study suggests that CXCR4 is not involved in the observed TIMP-1-induced inflammatory effects. However, our data do not fully rule out the possibility that CXCR4 may form a heteromeric receptor complex with CD74 to trigger TIMP-1 signaling. The lack of an inhibitory effect of AMD3100 on the pro-chemotactic activity of TIMP-1 at first sight suggests that TIMP-1 does not interact with CXCR4, but it may also be explained by the notion that the AMD3100 binding site on CXCR4 does not considerably overlap with the binding site of TIMP-1. Overall and in conjunction with our in silico and biochemical data, a lack of involvement of CXCR4 in TIMP-1-driven monocyte migration responses is the more-likely explanation. In one study, TIMP-induced signals were shown to be dependent on the formation of a ternary complex between TIMP-1, proMMP-9, and CD44 at the cell surface [64]. Therefore, it is in principle conceivable that CD44 may be involved, but further studies that are beyond the scope of this project are needed to answer this question.

Our study revealed that the effects induced by TIMP-1–CD74 exhibit similarities and comparability with those elicited by MIF–CD74. Still, it is currently unclear whether there exists a synergistic or counter-regulatory relationship between the effects mediated by the MIF–CD74 and TIMP-1–CD74 axes and also whether there is competition between MIF and TIMP-1 for CD74. One study performed by Onodera and colleagues previously reported that MIF can influence the mRNA levels of TIMP-1 in a dose-dependent manner in rheumatoid arthritis synovial fibroblasts [65]. Nevertheless, our investigation in monocytes did not reveal any significant effects of TIMP-1 on MIF and its receptors. Further investigations are certainly required to shed light on the interplay between MIF- (or MIF-2-) and TIMP-1-driven signals through CD74.

The impact of TIMP-1 on monocyte migration and adhesion, as well as VSMC proliferation, mediated through CD74, argue for its atherogenic properties. This notion was further supported by re-analysis of scRNA-seq datasets from the atherosclerotic plaque tissue of atherosclerotic patients. The analysis was performed on datasets from three different studies and revealed an elevated expression of both TIMP-1 and CD74, most prominently monocytes/macrophages and VSMCs. Intriguingly, the expression profiles of TIMP-1 in individual cell populations were distinct from those of CD74. Across all analyzed datasets, TIMP-1 expression was most-pronounced in MΦ-I (characterized by elevated expression of *Spp1*, *Fabp5*, and *Cstb*), monocytes, and VSMCs, but low in MΦ-II (with the signature genes *C1qa*, *Ms4a6*, and *Hla-Dqa1*). In contrast, CD74 was highest in MΦ-II and exhibited only minor expression in VSMCs. Together, this might suggest an inter-cellular communication axis between MΦ-I- and VSMC-derived TIMP-1 and CD74-expressing MΦ-II cells as responder cells. The expression profile of MIF was more similar to that of TIMP-1 than CD74, insinuating that it may feed into the same inter-cellular communication axis.

Previous studies have shown that elevated levels of TIMP-1 are associated with an increased risk of atherosclerosis and CVD [66,67,68,69,70]. However, the mechanistic role of TIMP-1 in the pathogenesis of atherosclerosis remains complex and controversial. In one study, Silence and colleagues showed that *Timp-1* deficiency in *Apoe^−/−^* mice was associated with a reduction of the size of atherosclerotic lesions, but with an increased accumulation of lesional macrophages and foam cells [71]. However, in another study, overexpression of TIMP-1 in *Apoe^−/−^* mice also resulted in a reduction of atherosclerotic lesions in the aortic root [72], whereas a similar study performed by Cuaz-Perolin et al. showed that overexpression of TIMP-1 did not affect the size of atherosclerotic plaques in *Apoe^−/−^* mice [73]. The discrepancy regarding the involvement of TIMP-1 in the pathogenesis of atherosclerosis observed in those studies may be related to its dual activity, acting both as an MMP inhibitor and as a cytokine/chemokine-like mediator. The combination of functional in vitro experiments and analysis of single cell data from plaque tissue of atherosclerotic patients performed in our present study argues in favor of a pro-atherosclerotic role for TIMP-1, in particular via its cytokine/chemokine activity, harnessing the CD74 receptor pathway.

Furthermore, previous studies performed in *Timp-1^−/−^Apoe^−/−^* mice have observed that the loss of TIMP-1 expression was associated with a reduction in smooth muscle cell content in atherosclerotic plaques, particularly in the aortic root [74] and the brachiocephalic artery [75]. Similarly, the deficiency of CD74 in *Ldlr^−/−^* mice has been associated with a protective effect against atherosclerosis [76], and CD74 has been found to be expressed in human atherosclerotic plaques, particularly in vascular cells [77] and apoptotic macrophages [78], contributing to plaque progression and clinical manifestation. We showed that TIMP-1 and CD74 are abundantly expressed in plaque monocytes/macrophages and that TIMP-1 is abundant in VSMCs. We also provided evidence that the signaling pathways activated by TIMP-1–CD74 are involved in regulating monocyte migration and adhesion and VSMC proliferation.

Next to confirming TIMP-1 as an emerging ligand for CD74 and comparing it with the *bona fide* ligand MIF, our study, to the best of our knowledge, presents novel evidence for a role of the TIMP-1–CD74 axis in atherogenesis by promoting monocyte migration and adhesion, as well as VSMC proliferation.

## 5. Conclusions

In summary, this study confirmed that TIMP-1 is a novel ligand of the cognate MIF receptor CD74 and showed, for the first time, that it also binds to the soluble CD74 variant sCD74. Importantly, our work provided novel insight into the involvement of the TIMP-1–CD74 axis in inflammatory/atherogenic monocyte recruitment responses and VSMC activation and identified PI3K and MAPK as downstream signaling pathways. In conjunction with the determined TIMP-1 and CD74 expression patterns in monocytes, certain macrophage sub-types, and VSMCs, as derived from the analysis of scRNA-seq datasets of plaques from atherosclerosis patients, these results, furthermore, imply that the TIMP-1–CD74 axis may play a significant role in the pathogenesis of atherosclerosis.

## Figures and Tables

**Table 1:** Comparative analysis of TIMP-1 and CD74 expression in monocytes, MΦ, and VSMCs revealed by analysis of scRNA-seq datasets of atherosclerotic tissues from three independent studies.

	TIMP-1 Expression			CD74 Expression		
	MO	MΦ	VSMCs	MO	MΦ	VSMCs
Alsaigh et al. [34]	++	++	+	++	+	−
Zernecke et al. [36]	++	++	+	++	+	−
Slenders et al. [35]	NA	+	++	NA	++	+

MO: monocytes; MΦ: macrophages; VSMCs: vascular smooth muscle cells; NA: not available.

## Data Availability

All data generated during the current study are contained within the manuscript and/or are available from the corresponding authors upon reasonable request.

## Supplementary Materials

The following Supporting Information can be downloaded at: https://www.mdpi.com/article/10.3390/cells12141899/s1, Table S1: Quantification of the 38 positive spots obtained from the phosphorylation kinase array in response to TIMP-1; Table S2: SpotON prediction of active residues in the TIMP-1/CXCR4 interaction; Figure S1: CXCR4 and CD74 are expressed in human pro-monocytic THP-1 cells; Figure S2: Re-analysis of three human atherosclerotic scRNA-seq datasets of two independent studies; Figure S3: Re-analysis of the scRNA-seq dataset of 38 human patients undergoing CEA; Figure S4: Highly expressed marker genes in the Mφ-I/II population of the atherosclerotic core in Alsaigh et al.’s scRNAseq dataset defining these populations.
