# Supplementary material for: Tissue Inhibitor of Metalloproteinases-1 Interacts with CD74 to Promote AKT Signaling, Monocyte Recruitment Responses, and Vascular Smooth Muscle Cell Proliferation
Source: Cells. 2023 Jul 20;12(14):1899. doi: 10.3390/cells12141899 (PMC10378328; doi:10.3390/cells12141899)
Supplement: Supplementary file 1 [file cells-12-01899-s001.zip › cells-2476795-supplementary.pdf]

Supplementary Materials

Article

Tissue Inhibitor of Metalloproteinases-1 Interacts with CD74 to Promote AKT Signaling, Monocyte Recruitment Responses, and Vascular Smooth Muscle Cell Proliferation

Simon Ebert [1], Lan Zang [2], Noor Ismail [1], Michael Otabil [1], Adrian Fröhlich [1], Virginia Egea [2], Susann Ács [2], Mikkel Hoeberg [3], Marie-Luise Berres [4], Christian Weber [2,5,6], José M. A. Moreira [3], Christian Ries [2], Jürgen Bernhagen [1,5,6,†] and Omar El Bounkari [1,*]

[1] Department of Vascular Biology, Institute for Stroke and Dementia Research, Klinikum der Universität München, Ludwig-Maximilian-University (LMU) Munich, 81377 Munich, Germany; juergen.bernhagen@med.uni-muenchen.de (J.B.)
[2] Institute for Cardiovascular Prevention (IPEK), Klinikum der Universität München, Ludwig-Maximilian-University (LMU) Munich, 80336 Munich, Germany
[3] Department of Drug Design and Pharmacology, Faculty of Health and Medical Sciences, University of Copenhagen, 2200 Copenhagen, Denmark
[4] Department of Internal Medicine III, RWTH Aachen University, 52074 Aachen, Germany
[5] Munich Cluster for Systems Neurology (SyNergy), 81377 Munich, Germany
[6] Munich Heart Alliance, 80802 Munich, Germany
* Correspondence: omar.el_bounkari@med.uni-muenchen.de
† Senior Author.

Supplementary Table S1: Quantification of the 38 positive spots obtained from the phosphorylation kinase array in response to TIMP-1 over time.

Target	Phosphorylation Site	10 min	30 min	60 min
**β-catenin**	--	102	93	116
**STAT-2**	Y689	104	102	97
**p38α**	T180/Y182	100	86	82
**JNK 1/2/3**	T183/Y185, T221/Y223	98	108	102
**Fgr**	Y412	102	101	100
**CREB**	S133	165	158	32
**STAT 5a/b**	Y694/Y699	92	88	83
**PDFG Rβ**	Y751	106	95	106
**Lck**	Y394	103	98	105
**GSK-3α/β**	S21/S9	24	23	135
**EGF R**	Y1086	100	96	98
**WNK1**	T60	21	46	21
**PLC-γ1**	Y783	104	92	97
**Lyn**	Y397	105	101	99
**GSK-3β**	S9	62	58	68
**eNOS**	S1177	237	95	101
**Yes**	Y426	102	95	93
**Src**	Y419	98	117	91
**MSK 1/2**	S376/S360	96	102	104
**HSP27**	S78/S82	128	127	99
**ERK 1/2**	T202/Y204, T185/Y187	81	114	77
**STAT6**	Y641	124	106	91
**STAT1**	Y701	102	98	97
**PYK2**	Y402	129	100	95
**p70 S6 kinase**	T389	111	96	100
**p53**	S15	111	103	101
**Chk-2**	T68	106	100	106
**Akt 1/2/3**	T308	121	99	102
**HSP60**	--	104	101	284
**STAT3**	Y705	108	97	99
**RSK 1/2**	S221/S227	108	99	104
**p70 s6 kinase**	T421/S424	100	117	100
**c-Jun**	S63	107	93	105
**Akt 1/2/3**	S473	114	447	107
**p53**	S392	113	127	99
**PRAS40**	T246	108	117	121
**RSK 1/2/3**	S380/S386/S377	96	95	127
**STAT3**	S727	106	111	140

Supplementary Table S2: SpotON prediction of active residues in the TIMP-1/CXCR4 interaction.

TIMP-1				CXCR4			
**Null Spots**		**Hot Spots**		**Null Spots**		**Hot Spots**	
PDB Nr.	AA	PDB Nr.	AA	PDB Nr.	AA	PDB Nr.	AA
1	CYS			35	ASN		
2	THR			151	LEU		
3	CYS			179	GLU		
4	VAL			180	ALA		
5	PRO			181	ASP		
6	PRO			182	ASP		
7	HIS			183	ARG		
10	THR			184	TYR		
14	ASN			185	ILE		
33	THR			187	ASP		
34	LEU			188	ARG		
36	GLN			190	TYR		
64	PRO			191	PRO		
65	ALA			192	ASN		
66	MET			193	ASP		
67	GLU			194	LEU		
68	SER			195	TRP	none	
69	VAL	none		196	VAL		
70	CYS			197	VAL		
73	PHE			201	PHE		
88	LYS			265	ILE		
90	GLN			266	LEU		
97	THR			267	LEU		
99	CYS			268	GLU		
129	VAL			269	ILE		
130	PHE			271	LYS		
131	PRO			273	GLY		
132	CYS			274	CYS		
133	LEU			275	GLU		
134	SER			277	GLU		
150	GLN			278	ASN		
151	LEU			281	HIS		
153	GLN						
154	GLY						
155	SER						

Supplementary Figure S1

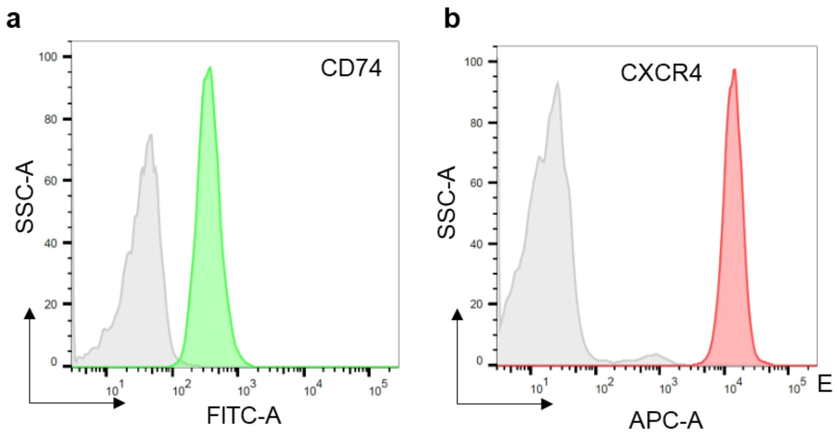

Figure S1: CXCR4 and CD74 are expressed in the human monocytic THP-1 cells. Cell surface expression of CD74 and CXCR4 in THP-1 cells was monitored by flow cytometry using FITC-conjugated anti-CD74 (**a**) (green) and APC-conjugated anti-CXCR4 (**b**) (red) or the corresponding APC-or FITC-conjugated IgG isotype controls (grey). Quantification of the obtained measurements was performed using FlowJo software.

Supplementary Figure S2

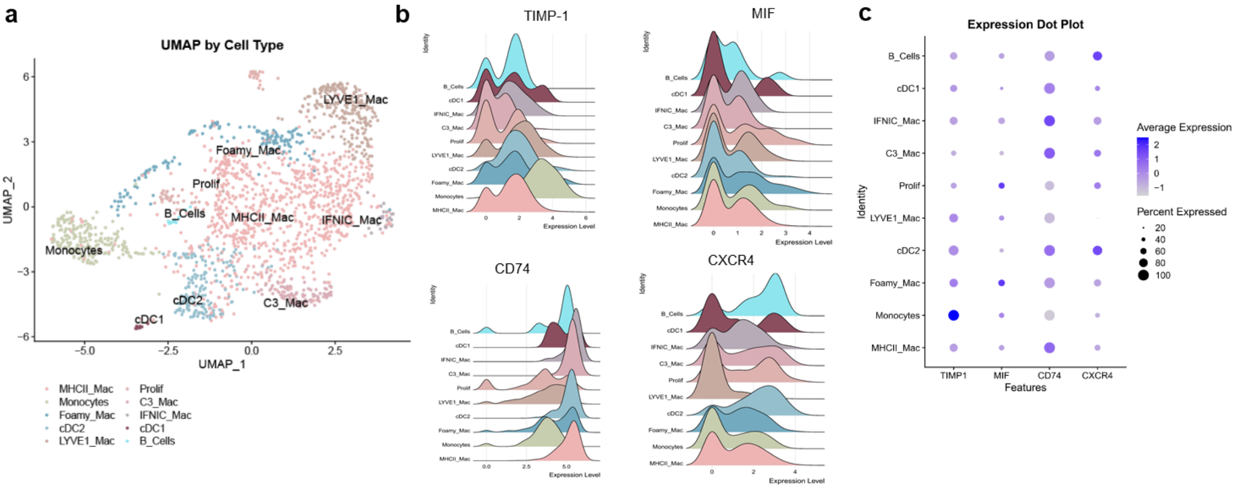

Figure S2: Re-analysis of three human atherosclerotic scRNA-seq datasets from two independent studies. Data were integrated by Zernecke et. al. and re-analyzed to examine the expression of TIMP-1, CD74, MIF, and CXCR4 in different single-cell populations using PlaqView [34,37]. (**a**) Uniform Manifold Ap-proximation and Projection of the integrated dataset with ten clusters identified by Louvain clustering. (**b**) Violin plots, and (**c**) dot plot of the genes of interest in the different cell populations found in the integrated scRNA-seq dataset. Xcr1^{+} Type1 classical DCs (cDC1), classical Cd209a+ DCs (cDC2), Macrophages (Mac), C3^{+} Macrophages (C3_Mac), and proliverating *TUBB*$^{+}$, *H2AFZ*$^{+}$, and *STMN1*$^{+}$ cells (Prolif.)

Supplementary Figure S3

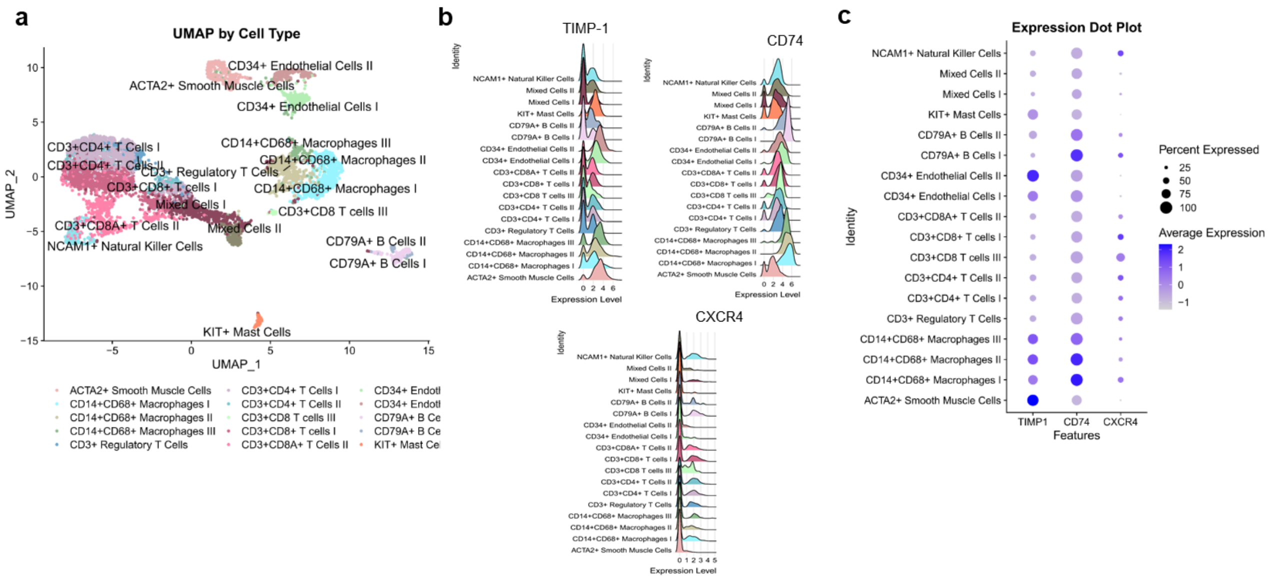

Figure S3: Re-analysis of the scRNA-seq dataset of 38 human CEA patients undergoing CEA [36]. Data were reanalyzed to examine the expression of TIMP-1, CD74, and CXCR4 in different single-cell populations using PlaqView [34,36].However, MIF was not found in this dataset. (**a**) Uniform Manifold Approximation and Projection of the integrated dataset with 18 clusters identified by Louvain clustering. (**b**) Violin plots, and (**c**) dot plot of the genes of interest in the different cell populations found in the integrated scRNA-seq dataset.

Supplementary Figure S4

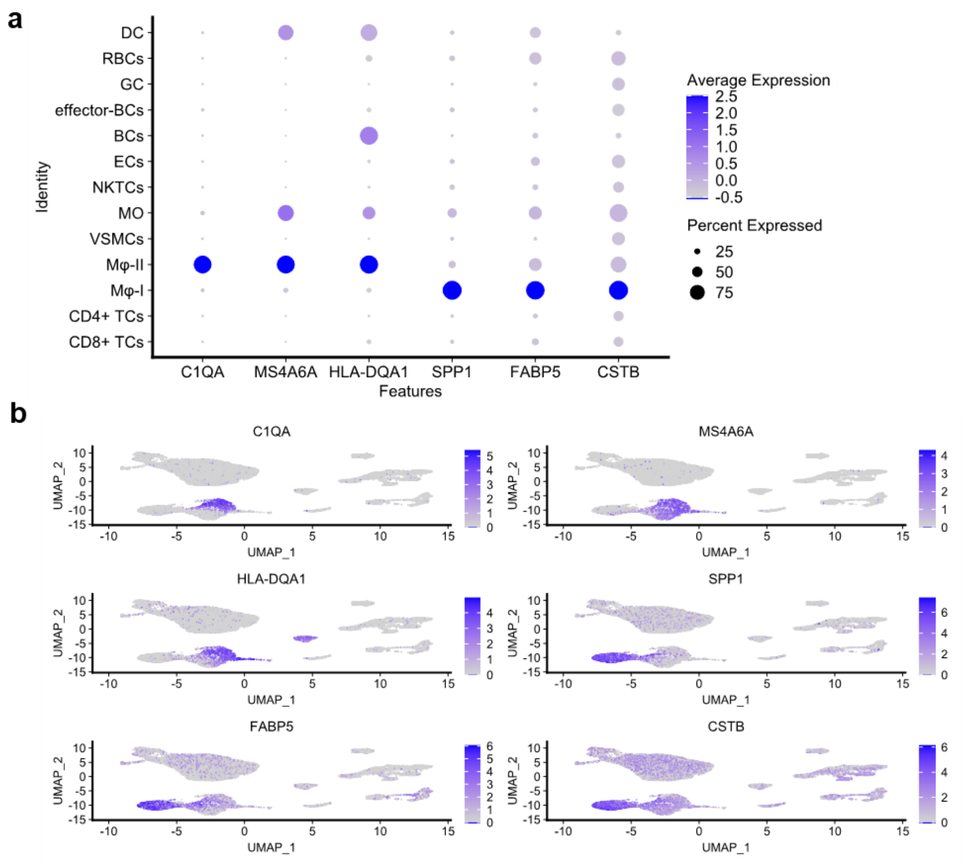

Figure S4: Highly expressed marker genes in the Mφ-I/II population of the atherosclerotic core in the Alsaight et al. scRNAseq dataset defining these populations [6]. (**a**) Dot plot visualization of the expression of *C1QA*, *MS4A6A*, and *HLA-DQA1* in Mφ-II macrophages, and *SPP1*, *FABP5*, and *CSTB* in Mφ-I macrophages. (**b**) Feature plot depiction of the spatial expression of Mφ-I/II marker genes in the UMAP, including B cells (BCs), T cells (TCs), natural killer T cells (NKTCs), red blood cells (RBCs), vascular smooth muscle cells (VSMCs), endothelial cells (ECs), granulocytes (GC), dendritic cells (DC), monocytes (MO), and macrophages (Mφ/MΦ).
